# High-Mobility Naphthalene Diimide Derivatives Revealed by Raman-Based In Silico Screening

**DOI:** 10.3390/ijms232113305

**Published:** 2022-11-01

**Authors:** Mikhail V. Vener, Oleg G. Kharlanov, Andrey Yu. Sosorev

**Affiliations:** 1 Kurnakov Institute of General and Inorganic Chemistry of the Russian Academy of Sciences, Leninskii Prosp. 31, Moscow 119991, Russia; 2 Faculty of Physics, Lomonosov Moscow State University, Leninskie Gory 1/2, Moscow 119991, Russia; 3 Shemyakin–Ovchinnikov Institute of Bioorganic Chemistry of the Russian Academy of Sciences, Miklukho-Maklaya St. 16/10, Moscow 117997, Russia

**Keywords:** periodic (solid-state) DFT calculations, low-frequency Raman spectrum, organic semiconductors, charge-carrier mobility calculations, dynamic disorder

## Abstract

Charge transport in crystalline organic semiconductors (OSCs) is considerably hindered by low-frequency vibrations introducing dynamic disorder in the charge transfer integrals. Recently, we have shown that the contributions of various vibrational modes to the dynamic disorder correlate with their Raman intensities and suggested a Raman-based approach for estimation of the dynamic disorder and search for potentially high-mobility OSCs. In the present paper, we showcase this approach by revealing the highest-mobility OSC(s) in two series of crystalline naphthalene diimide derivatives bearing alkyl or cycloalkyl substituents. In contrast to our previous studies, Raman spectra are not measured, but are instead calculated using periodic DFT. As a result, an OSC with a potentially high charge mobility is revealed in each of the two series, and further mobility calculations corroborate this choice. Namely, for the naphthalene diimide derivatives with butyl and cyclopentyl substituents, the estimated room-temperature isotropic electron mobilities are as high as 6 and 15 cm^2^ V^–1^ s^–1^, respectively, in the latter case even exceeding 20 cm^2^ V^–1^ s^–1^ in a two-dimensional plane. Thus, our results highlight the potential of using the calculated Raman spectra to search for high-mobility crystalline OSCs and reveal two promising OSCs, which were previously overlooked.

## 1. Introduction

Crystalline organic semiconductors (OSCs) with efficient charge transport, i.e., with high charge-carrier mobilities *μ* > 1 cm^2^ V^–1^ s^–1^, are a hot topic today, being necessary for the efficient operation of organic electronic devices [1,2,3]. The highest charge mobilities for organic semiconductors were observed in crystalline OSCs composed of π-conjugated molecules with fused heteroaromatic and/or aromatic cores [1,4]; nevertheless, they are low in comparison with the estimations of the physical limit for the intrinsic mobility, i.e., the one in the absence of defects, in OSCs (~70 cm^2^ V^–1^ s^–1^) [5]. Thus, the search for higher-mobility OSCs remains an urgent task for organic electronics. However, reliable measurement of the intrinsic *μ* in OSCs is not straightforward. For instance, a commonly used method based on fabrication of organic field-effect transistors with single crystals of the studied OSCs as active layers is complicated by the issues with the contacts, the architecture, the dielectric, etc. [6]. When polycrystalline films (which are much easier to process) of the OSCs are used as an active layer, the mobility is considerably (by over an order of magnitude) decreased by grain boundaries and other defects; moreover, relative mobilities for different OSCs do not necessarily represent the trends in the intrinsic mobilities [1,7]. Therefore, theoretical estimation of *μ* prior to its time-consuming measurements in devices is important for a focused search of high-mobility OSCs. However, though first-principle approaches are generally able to yield relatively accurate mobility predictions, they utilize a number of different frameworks and sometimes quite nontrivial approximations, which should be used with care (e.g., Boltzmann transport [8] versus Marcus hopping [9], or a bandlike motion of a small polaron [10] versus the semiclassical transient localization scenario [11]). Thus, such approaches are more suited for the final mobility estimations for shortlisted OSCs, while the initial screening step rather requires simpler, “blackbox-ready” approaches that allow for a rough *μ* estimation in a series of semiconductors and rely upon established computational techniques, such as the periodic DFT [1,2,3]. 

OSCs are “soft” materials—they consist of molecules bound by weak non-covalent forces. This softness affects the charge transport: the latter is hindered by significant modulation of the transfer integrals, *J*, between the molecules induced by thermally populated low-frequency (LF) vibrations with frequencies *ω* < 200 cm^–1^ [12,13,14,15,16]. Hereinafter, we use spectroscopic wavenumber units for the frequency (1 cm^–1^ standing for the frequency *ω* = 1.88 × 10^11^ s^–1^), with 200 cm^–1^ corresponding to the energy of thermal motion at 300 K. Physically, the “noise” in the transfer integrals, also referred to as the dynamic disorder, breaks the periodicity of the lattice and prevents the charge carrier from developing extended Bloch states, leading to (partial, transient) Anderson localization and thus suppressing the mobility [11,17,18]. Clearly, finding materials with a weak dynamic disorder is a promising route towards identification of high-mobility OSCs, and several strategies to improve the mobility in OSCs via reduction of the dynamic disorder have been suggested [5,19,20]. Indeed, though technically there is no *simple* relation between the characteristics of the dynamic disorder in a given OSC and the charge-carrier mobility (the one based on the Kubo formula [21] and the Holstein–Peierls model [22], as discussed in Section 3.4, should in no way be considered simple), the charge-carrier mobilities in OSCs reasonably correlate with such transparent quantities as the root mean square disorder amplitude *σ_J_* [19,23,24,25]. For example, a reasonable correlation with the experimental mobilities is expressed by an Einstein-like formula [19,23,24,25]:

(1) $$\mu \;\sim \frac{ea^{2}}{2k_{B}T}\;\frac{{\left(J/\sigma_{J}\right)}^{2}\;}{\tau_{\mathrm{in}}},$$

where *e* is the elementary charge; *a* is the lattice constant; *k*_B_ is the Boltzmann constant; *T* is the absolute temperature; and the transient localization time *τ*_in_ roughly corresponds to the characteristic periods of LF vibrations strongly modulating the transfer integrals *J*. The inverse square of the relative disorder, ${\left(J/\sigma_{J}\right)}^{2}$, in Equation (1) represents a typical scaling of the localization length of charge carriers around the room temperature [24,25,26]. In view of the existence of such correlations between *μ* and *σ_J_*, in what follows, we will mainly focus on the search of OSCs with a low dynamic disorder, in pursuit of high mobility.

The bottleneck of the *σ_J_* and hence the *μ* calculation is the estimation of the nonlocal electron–phonon interaction (NLEPI), which converts the thermal fluctuations of the structural parameters (intermolecular distances and molecular orientations) into the modulations of the transfer integrals and hence suppresses the mobility [27,28,29,30]. The strength of NLEPI can be quantified by the lattice distortion energy *L* [31,32,33], which is proportional to $\sigma_{J}^{2}$ in the low-frequency limit, and by the contributions of various vibrational modes to it, *L_i_* (for details, see Section 3.3). Very recently, we have suggested a Raman-based approach for estimation of NLEPI [24,31,34,35,36]. Specifically, we have shown that for LF vibrational modes, *L_i_* correlates with the Raman intensity of the mode, *I_i_*, yielding an estimate of the total lattice distortion energy:

(2) $$L\;\sim \;C_{\mathrm{LF}}\int_{\mathrm{LF}}\frac{I\left(\omega \right)}{\hbar \omega \left(n\left(\omega \right)+1\right)}d\omega ,$$

where I(ω) is the spectral Raman intensity (technically, the Raman scattering cross section per one OSC molecule); *ω* is the vibrational frequency (or the Raman shift); *n*(*ω*) is the population of the corresponding vibrational mode; and *ħ* is the Planck’s constant. The pre-factor *C*_LF_ = *C*_LF_(*J*, *α*, *E*_g_ − *ħω*_L_, *a*, …) is expected to depend on the molecular and crystal structure: the molecular polarizability *α*, the optical or HOMO–LUMO gap *E*_g_ minus the incident photon energy *ħω*_L_, the transfer integrals *J* and lattice constant(s) *a*, etc. [31]. Equation (2) was used in Ref. [31] mainly to uncover a clear correlation between the experimental Raman intensities of the LF vibrational modes and the calculated *L_i_*. In a subsequent work [24], an approach developing Equations (1) and (2) was tested on experimental Raman spectra, which showcased the efficiency of OSC screening based on the experimental Raman + in silico methodology. Note that contemporary Raman spectrometers enable reliable measurements down to ~5 cm^–1^ [37] and even below that, i.e., they can, in principle, detect all the Raman bands of most crystalline OSCs. A theoretical analysis of the physics underlying correlation (2) also revealed a reasonable scaling law of the pre-factor, *C*_LF_ ∝ (*E*_g_ − *ħω*_L_)^2/^*α*^2^ (which corresponds to the parameter *γ* = 1/2 in Ref. [24]). Note that in the classical, LF approximation, *n*(*ω*) + 1 ~ *k*_B_*T*/*ħω*, and Equation (2) can be simplified: *L* ~ *C*_LF_*I*_LF_/*k*_B_*T*, where $I_{\mathrm{LF}}=\int_{\mathrm{LF}}I\left(\omega \right)d\omega$ is the total Raman intensity in the LF range.

However, both the calculation and measurement of the absolute Raman intensity is not straightforward, with the spectral function I(ω) usually revealed up to a normalization constant (e.g., DFT packages typically use the Raman band with the highest intensity for this constant), thus, it appears advantageous to transform Equation (2) into an expression containing intensity *ratios* instead. It is convenient, for instance, to use a ratio of the LF Raman intensity to the “high”-frequency one (HF, *ω* > 200 cm^–^^1^). The latter is mainly associated with the electron–phonon interaction with intramolecular vibrational degrees of freedom, which is quantified by the reorganization energy, *λ*. This energy allows for an estimation $\lambda \;\sim \;C_{\mathrm{HF}}\int_{\mathrm{HF}}\frac{I\left(\omega \right)}{\hbar \omega }d\omega$ [24,38]; moreover, the physical analysis in Ref. [24] suggests a scaling *C*_HF_(*α*, *E*_g_ − *ħω*_L_, *a*, …) ∝ 1/*α*^2^ (which corresponds to the off-resonant case $\bar{\gamma }=1$ in Ref. [24]). Now, by dividing Equation (2) over the estimation for *λ*, one can eliminate the overall normalization of the Raman intensity in favor of a ratio of the LF intensity to the (weighted) HF one:

(3) $$L\propto \frac{\lambda {\left(E_{g}-\hbar \omega_{L}\right)}^{2}}{k_{B}T}R,R=\int_{\mathrm{LF}}\;I\left(\omega \right)d\omega /\int_{\mathrm{HF}}\;\frac{I\left(\omega \right)}{\hbar \omega }d\omega .$$

For compounds with a similar chemical structure—including the naphthalene diimide (NDI) derivatives studied in the present paper, see below—reorganization energies are of the same order (see, e.g., Figure 6 in Ref. [34] and Table S7 (Supplementary Information)); the same applies to the energy gaps. Thus, *R* can be used for estimating *L* and thus the dynamic disorder amplitude *σ_J_* (for details, see Refs. [24,36]): at least, the higher *R*, the higher *L* is expected for given *λ* and *E*_g_. The value of *R* can be estimated both experimentally and computationally. In the present study, we test a Raman-based in silico approach for the search of high-mobility crystalline OSCs, relying upon the Raman intensities calculated by periodic DFT.

A good benchmarking set for such tests is a series of OSCs differing in the alkyl chains end-capping the same π-conjugated unit [39,40]. For instance, NDI derivatives with alkyl and cycloalkyl terminal substituents have been synthesized, with the (polycrystalline) thin-film OFET charge-carrier mobilities measured for them [2,41,42,43]. Some of these compounds have shown high (close to record) electron mobilities, for instance, crystalline N,N′-dicyclo-hexyl-naphthalene-1,8;4:5-dicarboximid (**NDI-CHex**) [41] and N,N′-bis(hexyl)naphthalene diimide (**NDI-Hex**) [42]. However, charge-carrier mobility data for single crystals (i.e., estimates of the intrinsic mobilities) are lacking for most of the NDIs, so intrinsic mobilities for these OSCs are unknown. Thus, application of the Raman-based estimation of the dynamic disorder and calculation of single-crystal charge mobilities for various NDIs can reveal previously overlooked high-mobility OSCs.

**Figure 1:**
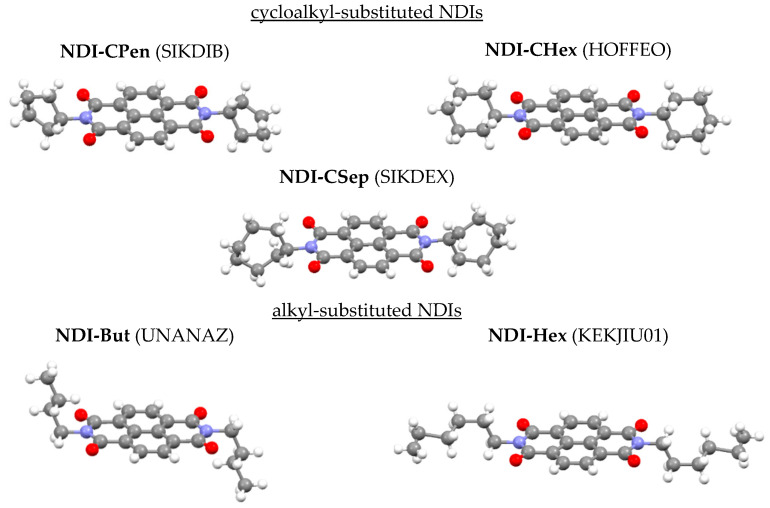

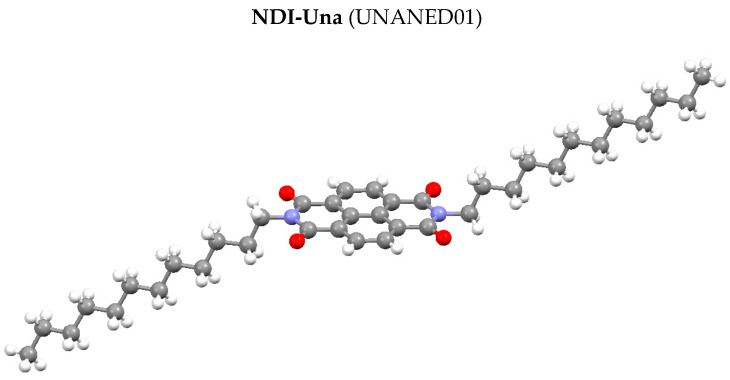
Fragments (primitive cells) of the crystals under study. Ref. codes (six-character IDs) of the crystals in the Cambridge Structural Database [46] are given in parentheses. The red, blue, and gray balls represent the oxygen, nitrogen, and carbon atoms, respectively, while the small white balls indicate the hydrogen atoms.

Six alkyl- and cycloalkyl-substituted NDIs are considered in this work, including the abovementioned **NDI-CHex** and **NDI-Hex**, see Figure 1. We evaluate the Raman spectra of these crystals using periodic DFT, with special attention paid to the accuracy of the calculated wave numbers and Raman intensities for the lowest-frequency Raman-active vibrations.

We find that the lowest *R* ratios (expected to correspond to the highest charge-carrier mobilities) are observed for NDIs with butyl (**NDI-But**) and cyclopentyl (**NDI-CPen**) substituents in the considered family of OSCs. We then proceed with an in-depth investigation of NLEPI in the six crystals and calculate the charge-carrier mobilities (including the anisotropy and temperature dependencies) within the transient localization scenario [11,19]. Note that, in the context of the present work, the role of the mobility calculations is twofold: first, they test the overall performance of the *R*-based in silico mobility screening approach suggested; second, they yield *quantitative* mobility predictions for the two shortlisted NDIs—beyond the *qualitative* conclusion of them being the best ones in the considered series. Namely, our analysis corroborates that these OSCs can show high room-temperature electron mobilities: about 13 cm^2^ V^–1^ s^–1^ along the high-mobility axis of **NDI-But** and around 23 cm^2^ V^–1^ s^–1^ along two axes in **NDI-CPen**. These values exceed that for the previous record-breaker **NDI-CHex**, while the one for **NDI-CPen** even potentially beats the figure for rubrene [44] and, as we further verify, is quite robust against the so-called “killer mode”, dropping only to 10–15 cm^2^ V^–1^ s^–1^ in supercells supporting this mode [45]. Thus, our results both highlight the potential of using the Raman spectrum for screening of high-mobility crystalline OSCs among compounds with similar chemical structure and suggest a potentially record-high-mobility material, **NDI-CPen**, relating its expected properties to the Raman spectrum.

## 2. Results and Discussion

### 2.1. Raman Spectra of NDI Derivative Crystals, the R Ratios, and Dynamic Disorder

The lowest frequency Raman-active vibrations of single-component organic crystals are often characterized by relatively low Raman-scattering intensities, namely, ~5% of the most intense band [47,48,49,50,51]. Thus, errors in the calculation of wavenumbers (up to 10 cm^–1^) and/or of the Raman intensities (up to two times) of such modes do not play a significant role in the *R* calculation. In contrast to the general case of organic crystals, however, in OSCs [31,52] and in some multicomponent organic crystals [53,54], the lowest frequency Raman-active vibration can be very intensive; moreover, it can be located around or below 10 cm^–1^ [55,56]. For such a mode, errors reaching 10 cm^–1^ in the calculation of the wavenumber, caused by basis set changes (see Tables S1 and S2 in Ref. [31]) or by the choice of the functional (see Tables S1 and S2), which are observed for the OSCs studied, can radically change the frequency of the mode. Note that 10 cm^–1^ is also a typical value of the Lorentzian broadening used when comparing vibrational bands with experimental ones, see, e.g., [57]. Moreover, the calculated Raman intensity of the lowest-frequency band can also differ significantly from the experimental value (see Figure S3 in Ref. [58]). Such discrepancies may be due to the limited applicability of the “double harmonic approximation” [59] for the quantitative description of the Raman spectra of organic crystals below 20 cm^–1^. For these reasons, in the following, the Raman-active bands below 20 cm^–1^ are treated with care, and their DFT predictions are not considered as ultimate.

In this regard, especially since LF vibrational modes are concerned, it is worth making some general remarks on the accuracy of DFT calculations of Raman spectra of OSCs, before switching to a full analysis of the six NDI crystals. We start with the calculations of the Raman spectra of crystalline **NDI-Hex** and **NDI-CHex**, for which experimental data are available [31], using several functionals and the 6-31G** basis set, in order to choose the one that fits the data best. A comparison between the calculated and experimental spectra is given in Tables S1 and S2. The PBE-D3/6-31G** level gives a better description of the spectra compared to PBEsol/6-31G** and B3LYP/6-31G**, in line with our earlier findings [53]. In particular, we are able to conclude that the wavenumber accuracy for the LF Raman-active bands of the two crystals calculated using PBE-D3 is acceptable: e.g., for the modes above 20 cm^–1^, the calculations agree with experiment within 5%.

**Figure 2:**
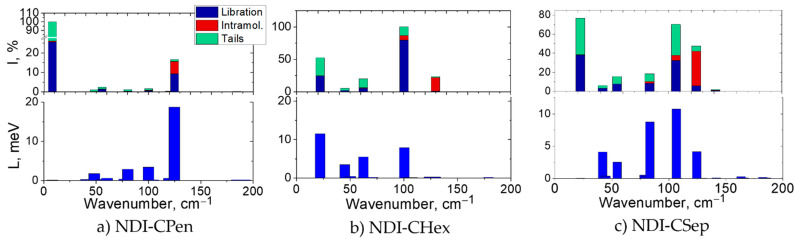
Computed LF Raman spectra (top) and contributions of various vibrational modes to NLEPI in terms of the lattice distortion energy *L* (bottom) for the considered crystals with cycloalkyl substituents. In the top panels, contributions from various types of motion (librational and intramolecular vibrational motions of the NDI cores, as well as substituent, “tail” motions) to the vibrational modes are shown.

**Figure 3:**
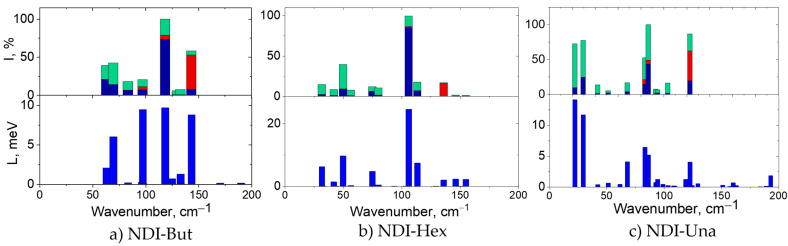
Computed LF Raman spectra (top) and contributions of various vibrational modes to NLEPI in terms of the lattice distortion energy *L* (bottom) for the considered crystals with alkyl substituents. In the top panels, contributions from various types of motion (librational and intramolecular vibrational motions of the NDI cores, as well as substituent, “tail” motions) to the vibrational modes are shown (coloring corresponds to Figure 2).

With the above in mind, we now present the Raman spectra of the six studied OSCs calculated using periodic DFT at the PBE-D3/6-31G** level in Figure 2, Figure 3 and Figure S1 . Their common features are: (i) the most intense Raman band lies in the LF region of the spectrum; (ii) intense Raman bands are practically absent in the 200–500 cm^–1^ range, in line with Refs. [31,51,54,55] (we use the term “intense band” for vibrations having relative Raman intensities of more than 5%, compared with the most intense band hereinafter normalized to 100%); (iii) the Raman spectra of the crystals under consideration are very similar in the HF range (with the only exception in the range around 3000 cm^–1^ related to CH vibrations of the substituents), which can be readily explained by the similarity of their conjugated cores, in accord with the literature data (see Figure 6 in Ref. [34]). Thus, the specific features of our crystals manifest themselves in the LF range of the Raman spectrum.

The cycloalkyl NDIs have around 5 intense Raman bands in the LF range (see Figure 2 and Tables S2 and S3). Such spectra, with less or around 5 intense Raman bands in the LF range will be called relatively “sparse” below. The number of intense bands in the LF region of the Raman spectra of the alkyl-substituted NDI crystals is greater than that for the NDIs with cyclic substituents (see Figure 3 and Tables S1–S3), reaching 12 for **NDI-Una**. This could be related to a softer character of the alkyl chains compared with cycloalkyls; moreover, with the increase in the substituent length, the number of vibrational degrees of freedom also increases.

In the NDIs with cycloalkyl substituents, the LF modes with the highest Raman intensities are located below 25 cm^–1^ and around 100 cm^–1^ (see Figure 2 and Tables S2 and S3). The atomic displacements for the former modes in these crystals are mainly composed of librations (rotations) of the NDI core around the *short axis* of the molecule, accompanied by motions of the cycloalkyl substituent (see Table S4; see also Figure 3c in Ref. [31]). The displacements of the atoms of the cyclic substituents are even larger than those of the NDI core atoms in this vibrational mode. In contrast, the higher-frequency modes around 100 cm^–1^ with dominant Raman intensities correspond mainly to librations of the NDI core around the *long axis* of the molecule (see Table S4; see also Figure 3e in Ref. [31]). Notably, the atomic displacements of the cyclic substituents are practically negligible in these modes.

In the alkyl-substituted NDIs, the most intense Raman band is located near 100 cm^–1^ (see Figure 3 and Tables S2 and S3), and the wavenumber of this band in **NDI-But**, 118 cm^–1^, is the highest in the series. Moreover, the wavenumber of the lowest-frequency Raman-active vibration in **NDI-But** (63 cm^–1^) is more than twice the corresponding wavenumbers for crystalline **NDI-Una** and **NDI-Hex**. Analogously to the cycloalkyl-substituted NDIs, it is possible to single out Raman-intense modes in which the atomic displacements correspond mainly to librations (rotations) of the NDI core around the short axis of the molecule and around its long axis (see Table S4 and also Figure 3d,f in Ref. [31]).

Contributions of librational motions, intramolecular vibrations of the conjugated core, and motions of the (cyclo)alkyl substituents to the vibrational modes are shown in Figure 2 and Figure 3. Unsurprisingly, the lowest-frequency Raman-active modes in the crystals studied are predominantly librational (see also Table S4). Indeed, Γ-point vibrational modes of a crystal with *Z*_red_ = 1 do not include a translational component, i.e., the centers of mass of the molecules remain at rest. For a perfectly rigid molecule, the residual motion is inevitably rotational, i.e., corresponds to librations around a certain axis. Now, our molecules have a rigid enough condensed aromatic core (which is even more rigid under low-frequency perturbations), attached to flexible (cyclo)alkyl substituents. Thus, it is quite natural that LF Γ-point vibrational modes in Figure 2 and Figure 3 consist of sizeable NDI core librations accompanied by mixed librational/buckling motions of the substituents. Notably, the atomic displacements for librational modes are antisymmetric (odd) with respect to the rotation center, i.e., such modes are Raman-active. The strong Raman signal for these modes (and the strong coupling of these modes to the intermolecular transfer integrals, see below) are expectable, since the corresponding vibrations alter relative orientations of the NDI cores and, hence, the polarizability of the crystal [31]. Strictly speaking, an exception from the above picture is the mode around ~125 cm^–1^ with a considerable intramolecular contribution in all the crystals studied. However, a closer look reveals that this contribution also stems mainly from librations of the central segment of the NDI core around the long axis, which are not synchronized with those of the core edges, leading to its torsional deformation (see Table S4).

Finally, the LF/HF ratios, *R*, obtained from the calculated Raman spectra of NDIs using Equation (3), are summarized in Table 1. In view of the inaccuracies of DFT-calculated Raman wavenumbers and intensities of the lowest-frequency bands discussed above, we additionally estimated the *R* ratio neglecting the mode below 20 cm^–1^ (such a mode is present only for **NDI-CPen**). According to these findings, there are two crystals that are worth particular attention. The first one is **NDI-But**, which has the smallest *R*. The second one is **NDI-CPen**, which has the smallest *R* if the mode at 8 cm^–1^ is not taken into account. 

Namely, as discussed in Refs. [31,34], the small *R* values in these crystals should indicate a weak dynamic disorder (and hence, potentially high charge-carrier mobilities) therein. Below, we will analyze how the *R* values correlate with the dynamic disorder and present the evidence that **NDI-But** and **NDI-CPen** are indeed promising for organic electronics.

### 2.2. Nonlocal Electron-Phonon Interaction

In order to analyze the expected correlation between the computed Raman spectra and the strength of the dynamic disorder (or, equivalently, the lattice distortion energy *L*, see Equations (4) and (5)), in Figure 2 and Figure 3 we present the contributions of various vibrational modes $L_{i}=\sum_{mn}L_{i,mn}$ to *L* and collate them to the corresponding Raman intensities for the crystals studied. As follows from these figures, all the modes with considerable *L_i_* values are Raman-active, in line with Ref. [31] and with the above discussion of the Γ-point modes in Section 2.1. Moreover, the contributions of the modes to *L* correlate with their Raman intensities, also in line with Ref. [31], except for the 97 cm^–1^ mode in **NDI-But** having a low Raman intensity but a high *L_i_*, and the lowest-frequency modes in **NDI-CPen** and **NDI-CSep**, which have very high Raman intensities but weakly contribute to *L*. As discussed in Section 2.1, the discrepancy between *L_i_* and *I_i_* for the lowest-frequency vibrational modes could originate from their potentially inaccurate treatment within the used computational methodology.

Being convinced by Figure 2 and Figure 3 that Raman spectrum reveals (to some extent) the contributions of different vibrational modes to dynamic disorder, we turn to checking the correlation between the LF/HF ratio *R* and the relative dynamic disorder amplitude quantified as σ*_J_*/*J* (for the definition of the latter quantity, see Section 3.3). The data for *R*, *L*, and σ*_J_*/*J*, as well as the equilibrium *J* values, are listed in Table 1; additionally, this table presents the ratios $\tilde{R}=\lambda {\left(E_{g}-\hbar \omega_{L}\right)}^{2}R/k_{B}T$ expected to correlate with *L* (see Equation (3)). According to the table, the lowest *R* values are observed for **NDI-But** and **NDI-CPen** (for the latter one, neglecting the first Raman-active mode); Table 1 also shows that these crystals demonstrate low disorder amplitudes. Indeed, as follows from the NLEPI details in **NDI-But** and **NDI-CPen** given in Tables S5 and S6, the disorder in *J* is moderate in both crystals: in **NDI-But**/**NDI-CPen**, *σ_J_* amounts to 0.2*J*/0.21*J* in the direction with the largest *J* (the **a/c** crystal axis) and to 0.31*J*/0.15*J* in the direction with the second-largest *J* (the **b/a** axis). An overall correlation between the LF/HF ratios (both *R* and $\tilde{R}$) and *σ_J_*/*J* is also vividly shown in Figure S2. One should admit that the correlation between *R* and the *relative* disorder (*σ_J_*/*J*) is partially masked by the large transfer integrals, reaching 135 meV in **NDI-But** and 113 meV in **NDI-CPen** (cf. the values *J* = 118 meV in rubrene [60] and *J* = 69 meV in F_2_-TCNQ [61]—the OSCs showing a record hole mobility and one of the highest electron mobilities reported to date, respectively [1]). However, Table 1 and Figure S2 show that *R* also correlates with the *absolute* disorder amplitude $\sigma_{J}\propto \sqrt{L}$ within each of the two NDI series. Apart from that, another spectral quantity is worth pointing out—the number of intense enough Raman bands in the LF range—and Table 1 demonstrates that this quantity correlates quite well with *σ_J_ for the whole set of six NDIs*. In any case, based on our analysis, it can be thus expected that (i) **NDI-But** and **NDI-CPen** crystals are characterized by potentially high electron mobilities in the considered series of alkyl- and cycloalkyl-substituted series of OSCs, respectively, and (ii) the quantities *R*, $\tilde{R}$ (and the number of intense Raman bands) can be used for the screening of high-mobility OSCs. Both these statements are further justified by direct charge transport calculations in the following section.

### 2.3. Charge Transport Characteristics

We finally turn to charge-transport calculations in all the studied NDI derivatives within the transient localization framework (for details, see Section 3.4, and Refs. [11,19,31,62]). As mentioned in Section 1, in the context of the present work, these calculations pursue two goals. First, they are meant to additionally verify our Raman-based screening methodology by comparing the *qualitative* trends in *R* and the calculated mobilities (which is potentially important for OSCs in general beyond the two NDI series considered here). Second, after the Raman-based analysis of the previous sections has shortlisted two NDI crystals in these series, **NDI-But** and **NDI-CPen**, the calculations are expected to yield *quantitative* (albeit approximate) predictions of the electron mobilities in them, in cm^2^ V^–1^ s^–1^.

**Figure 4:**
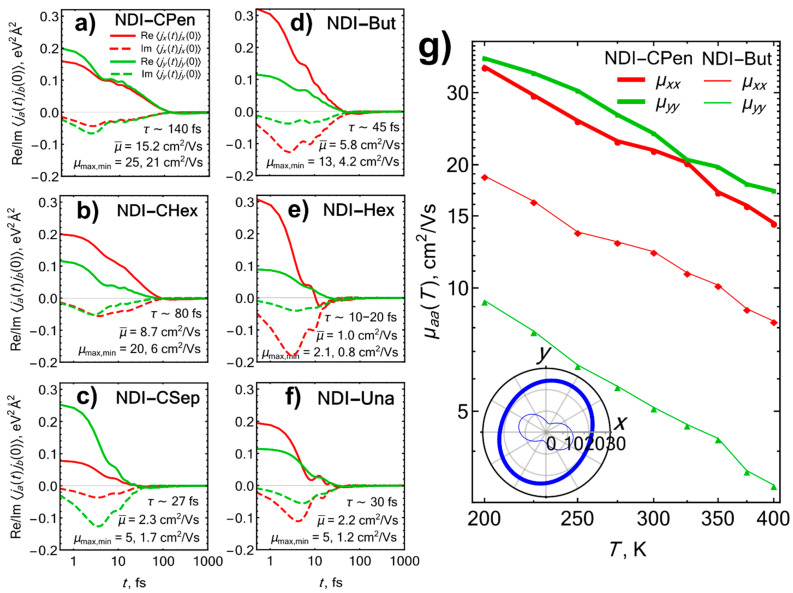
Electron transport properties in the slab plane (*xy*) of NDI derivatives: room-temperature current correlators $\langle {\hat{j}}_{x}(t){\hat{j}}_{x}(0)\rangle$, $\langle {\hat{j}}_{y}(t){\hat{j}}_{y}(0)\rangle$ evaluated within the transient localization scenario (**a**–**f**) and temperature dependences of the mobility in NDI-CPen and NDI-But (**g**). In panels (**a**–**f**), solid/dashed lines show the real/imaginary parts of the correlators; $\mu_{\max,\min}$ are the maximum and the minimum mobility values in the slab plane, respectively, and $\bar{\mu }=(\mu_{xx}+\mu_{yy})/3$ is the mobility averaged over all directions in 3D. These panels also present the characteristic times *τ*, at which the real parts of the correlators cross zero, as a qualitative estimation of the correlation decay rates. Thick and thin blue lines in the inset in panel (**g**) represent the mobility anisotropies in the slab planes of NDI-CPen and NDI-But, respectively, with the figures given in cm^2^ V^–1^ s^–1^.

Note that for the first of the above goals, it can be instructive to evaluate not necessarily the mobility itself, but also other, more qualitative characteristics relevant to charge transport. For instance, by virtue of the Kubo formula (6), the mobility at a given temperature is proportional to a time integral of the real part of the charge current correlator $\langle {\hat{j}}_{a}(t){\hat{j}}_{b}(0)\rangle$, so the profile of the latter can provide signatures of efficient, coherent charge transport in organic crystals [11,19,31]. In particular, the very characteristic correlation decay time *τ* provides an estimation of the coherence time of charge carriers and, in turn, is able to benchmark their mobility [31]. Thus, along with the calculated mobility values, in Figure 4a–f, we have plotted the current correlators for the six NDI derivatives studied; the calculation has been restricted to the slab plane (*xy*), as the nonnegligible transfer integrals lie in it, virtually making the transport (at most) two-dimensional (see the discussion of slabs and layers in Section 3.1). One immediately observes that among the three cycloalkyl-substituted NDIs shown in Figure 4a–c, **NDI-CPen** demonstrates the longest-living correlations; the second-best in this regard is **NDI-CHex**, and **NDI-CSep** is the worst, which is well in line with the numbers of intense Raman bands, the relative dynamic disorder magnitudes *σ_J_*/*J*, and the LF/HF ratios *R* summarized in Table 1. The same applies to NDIs with alkyl substituents (Figure 4d–f): **NDI-But** is the winner here, characterized with both long-living correlations and their high amplitude along one of the axes (cf. a large transfer integral *J*_1_ and a small *J*_2_ in Table 1); this agrees with the best *R* ratio, the smallest number of Raman-intense LF bands, and the lowest relative dynamic disorder *σ_J_*/*J* in **NDI-But**, as compared with **NDI-Hex** and **NDI-Una** (see Table 1).

The mobility values resulting from time integration of the correlators also agree with the above semiqualitative analysis based on the correlation amplitudes and decay times. Figure 4a–f gives the eigenvalues $\mu_{\max},\;\mu_{\min}$ of the 2 × 2 mobility tensor in the slab plane (which are the maximum and the minimum values of the mobility in it, respectively), along with the “isotropic” mobility $\bar{\mu }=(\mu_{xx}+\mu_{yy})/3$ averaged over all directions in 3D space. Here, **NDI-CPen**, **NDI-CHex**, and **NDI-But** demonstrate the highest values, which agree with the first and the last ones being shortlisted from NDIs with cycloalkyl and alkyl substituents, respectively, via a Raman-based analysis. Thus, our results corroborate the suggestion that calculated Raman spectra can be used for assessment of the dynamic disorder and for in silico screening of high-mobility OSC materials.

As for the absolute, quantitative mobility predictions, our estimations are quite close to the $\bar{\mu }$ values available for **NDI-CHex** and **NDI-Hex** from both experimental [41] and theoretical [63] studies. For the two champions, **NDI-CPen** and **NDI-But**, our calculations demonstrate a pronounced band-like, inverse-power-law temperature dependence of the mobility, $\mu \left(T\right)\propto T^{-\gamma }$, with **NDI-But** also having a high mobility anisotropy (see Figure 4g and the inset in it); both these features are attested to be characteristic of high-mobility OSCs with a coherent transport [1]. Notably, for **NDI-CPen**, which is even poorer in Raman-intense vibrational modes than **NDI-(C)Hex**, the average electron mobility reaches a record-high value of 15 cm^2^ V^–1^ s^–1^ and the mobility values in the slab plane turn out to exceed 20 cm^2^ V^–1^ s^–1^ (Figure 4g), a characteristic figure for rubrene single crystals [44]. Finally, since it is known that the effect of the translational (sliding) “killer” mode observed in THz spectra dominates NLEPI and can drastically suppress the mobility in some OSCs [45], we estimated the electron mobility in **NDI-CPen**, starting from periodic DFT vibrational mode calculations in 1 × 2 × 1 and 2 × 1 × 1 supercells to access such modes. For both supercells, the mobility did drop down, but not drastically, to about 10–15 cm^2^ V^–1^ s^–1^, retaining a considerable degree of isotropy, even despite the preferred **a** or **b** axes resulting from the choice of the supercell (see Figure S3). Thus, with *two* directions supporting such mobility values, the predicted average mobility in **NDI-CPen** is still greater or equal to the figure for rubrene [40], for which the transport is virtually one-dimensional.

In addition, based on our calculations, we are able to conclude that the shorter the (cyclo)alkyl substituent within the series studied, the weaker the dynamic disorder (specifically, the smaller *σ_J_*/*J*) and the larger the charge-carrier mobility, and that cycloalkyl-substituted NDIs show larger *μ* values than their alkyl-substituted counterparts. Noteworthily, the predicted mobility trends differ from those observed in thin-film OFET measurements in Refs. [42,43]. As we mentioned in Section 1, this can be explained by the fact that in the latter case, charge mobility was limited by grain boundaries and other defects and could be very far from the intrinsic mobility. Thus, we suggest making careful charge mobility measurements in OFETs with **NDI-CPen** and **NDI-But** single crystals as the active layers: we anticipate these studies will corroborate high (probably record-breaking) intrinsic electron mobilities in these OSCs.

Summing up, in this paper, we propose a Raman-based in silico screening protocol for the search of high-mobility organic semiconductors, which consists of the following steps. First, an organic compound consisting of a π-conjugated unit with some substituents and characterized by a reasonably good mobility is considered as a starting point. Next, in the structural databases, compounds are searched with a similar chemical structure, that is, a similar π-conjugated unit and various substituents. Preference is given to the crystals that are characterized by one molecule per primitive cell, *Z*_red_ = 1. Finally, for these crystals, a Raman spectrum is calculated, and for the crystals demonstrating “sparse” LF Raman spectra and/or low LF/HF ratios, the mobility is evaluated using known methods.

## 3. Materials and Methods

### 3.1. Crystals under Study

We considered six NDI-core molecular crystals: dicyclopentylbenzo[lmn][3,8]phenanthroline-1,3,6,8(2H,7H)-tetrone (**NDI-CPen**) [43]; N,N′-dicyclo-hexyl-naphthalene-1,8;4:5-dicarboximid (**NDI-CHex**) [41]; 2,7-dicycloheptylbenzo[lmn][3,8]phenanthroline-1,3,6,8(2H,7H)-tetrone (**NDI-CSep**) [43]; and 2,7-dibutylbenzo[lmn][3,8]phenanthroline-1,3,6,8(2H,7H)-tetrone (**NDI-But**) [64]; N,N′-bis(hexyl)naphthalene diimide (**NDI-Hex**) [42]; 2,7-didodecylbenzo[lmn][3,8]phenanthroline-1,3,6,8(2H,7H)-tetrone (**NDI-Una**) [65]. Five crystals were described by space group $P\bar{1}$, molecules lay on inversion centers and *Z* = 1. One crystal (**NDI-CHex**) was described by space group *C*2/*m*, the molecule was located in the m plane, as well as on the perpendicular axis 2, *Z* = 2. All crystals were characterized by one molecule per primitive cell, *Z*_red_ = 1. Technically, we adopted a definition *Z*_red_ = Z/*N*, where *N* depended on the type of the Bravais lattice: *N* = 1 for primitive lattice *P*; *N* = 2 for centered *A*, *B*, *C*, and *I*; *N* = 3 for *R*; *N* = 4 for *F*. These crystals possess different types of the substituent in the aromatic ring, namely, cyclic or linear ones (Figure 1). **NDI-But** and **NDI-Una** crystals had n-butyl and n-dodecyl chains, respectively, while crystalline **NDI-CSep** and **NDI-CPen** had cycloheptyl and cyclopentyl substituents, respectively**.** The corresponding crystal structures were reported in Refs. [43,65]. The **NDI-CHex** crystal was monoclinic, and its space group was *C*2/*m*. The other five crystals were triclinic, with a $P\bar{1}$ space group. NDI cores of the considered crystals formed layers (see Figure 2 in Ref. [31]). In **NDI-CHex**, the layers lay in the *ac* plane (Figure S3 in Ref. [31]), while in the other five crystals they did not correspond to any crystal plane (Figure S4 in Ref. [31]). NDI cores from adjacent layers contacted each other and formed slabs which were parallel to the *ab* plane in **NDI-Chex**, **NDI-CSep**, **NDI-But**, **NDI-Hex**, and **NDI-Una**, or the *ac* plane in **NDI-CPen** (Figure 2 in Ref. [31]). All the crystals had inversion symmetry centers, so that they had two types of vibrational modes: only IR-active and only Raman-active ones. 

Note that the six crystals considered in this work (Figure 1) were characterized by *Z*_red_ = 1, unlike the crystals of NDI and naphthodithiophene diimide derivatives with *Z*_red_ = 2, for which electron mobility has recently been studied, both experimentally and theoretically [66,67].

### 3.2. Periodic DFT Calculations

In the CRYSTAL17 calculations [68], we employed the PBE [69], B3LYP [70,71], and PBEsol [72] functionals with the all-electron Gaussian-type localized orbital basis sets 6-31G**. London dispersion interactions were taken into account by using the semi-empirical D3 scheme [73]. PBE-D3 and B3LYP gave a reasonable description of the LF Raman spectrum of organic crystals in periodic DFT calculations [31,51,53,74,75], while PBEsol was used to study the energetics of molecular crystals [76]. The space groups and the unit cell parameters of the crystals obtained from the X-Ray diffraction experiment were fixed and the structural relaxations were limited to the positional parameters of the atoms [53]. This approximation was widely used in periodic DFT calculations of various properties of organic crystals [77,78,79,80]. Raman intensities were calculated using the “RAMANEXP” keyword of CRYSTAL17 software. The temperature was set to 298 K, the wavelength of the incident laser light 633 nm.

For DFT calculations aimed at estimating the impact of the “killer mode”, 2 × 1 × 1 and 1 × 2 × 1 supercells were used, formed by doubling the experimental ones. Further details are given in Section S1.

### 3.3. Evaluation of Electron-Phonon Couplings, Energy Gaps, and Reorganization Energies

The transfer integrals $J_{mn}$ between molecular sites *m*, *n* were calculated using the dimer projection method (DIPRO) [81]. Nonlocal electron-phonon couplings $\partial J_{mn}/\partial Q_{i}$, where $Q_{i}$ is the dimensionless vibrational coordinate corresponding to LF mode *i*, were calculated as the differences between the $J_{mn}$ values in equilibrium and with the atomic displacements corresponding to $Q_{i}=1$ along the normal modes from the periodic DFT calculations. The contribution of vibrational mode *i* with frequency *ω_i_* to the lattice distortion energy in the corresponding direction *mn*, $L_{i,mn}$ [82], was calculated as:

(4) $$L_{i,mn}=\frac{1}{2\hbar \omega_{i}}{\left(\frac{\partial J_{mn}}{\partial Q_{i}}\right)}^{2},$$

and the overall lattice distortion energy in this direction was calculated as $L_{mn}=\sum_{i}L_{i,mn}$. The total lattice distortion energy, which includes the contributions from various charge-transfer directions (*mn*), was obtained as $L=\sum_{mn}L_{mn}=\sum_{i,mn}L_{i,mn}$. The variance of the transfer integrals, which quantifies the dynamic disorder, reads [33]:

(5) $$\sigma^{2}\left(J_{mn}\right)=2k_{B}TL_{mn}$$

In line with our previous studies [24,35,36], we estimated the relative standard deviation of the transfer integrals as $\sigma_{J}/J=\sqrt{\sum_{mn}^{}\sigma^{2}\left(J_{mn}\right)}/\sqrt{\sum_{mn}^{}J_{mn}^{2}}.$ For the mobility calculations, we also evaluated the local electron–phonon couplings, $\partial \varepsilon_{m}/\partial Q_{i}$, where $\varepsilon_{m}$ are the LUMO energies [33]. These couplings were estimated as the differences between the $\varepsilon_{m}$ values in equilibrium and with the atomic displacements corresponding to $Q_{i}=1$ along the LF vibrational modes. For the evaluation of $\varepsilon_{m}$ and $J_{mn}$, non-periodic (single-molecule and dimer) DFT calculations at the PBE/6-31G** level were performed in the GAMESS package [83,84]. Reorganization energies were calculated using the four-point scheme [33]. Optical gaps were calculated at the B3LYP/6-31G** TDDFT level.

### 3.4. Evaluation of Charge-Carrier Mobility

The charge-carrier mobility tensor was evaluated using the Kubo formula [21]:

(6) $$\mu_{ab}\left(T\right)=\frac{e}{2k_{B}T}\int_{-\infty }^{\infty }\;\langle {\hat{j}}_{a}\left(t\right){\hat{j}}_{b}\left(0\right)\rangle dt,\hat{j}\left(0\right)=-\frac{i}{\hbar }\sum_{m,n}\;x_{mn}\left[J_{mn}+\sum_{i}\;\left(\partial J_{mn}/\partial Q_{i}\right)Q_{i}\right]{\hat{c}}_{m}^{†}{\hat{c}}_{n}.$$

In this formula, *a*, *b* refer to Cartesian axes in the *slab* plane (see Section 3.1), *m*, *n* to molecular sites in a 20 × 20 × 1 “transport supercell” with periodic boundary conditions, and *i* to vibrational modes on all the sites in this supercell. The operator ${\hat{c}}_{n}$ annihilates an electron on site *n* and $x_{mn}$ is a vector connecting two sites. To yield the charge current correlators $\langle {\hat{j}}_{a}(t){\hat{j}}_{b}(0)\rangle$, the time evolution of current operators $\hat{j}$ and a further canonical-ensemble averaging were computed with respect to the Holstein–Peierls Hamiltonian (see, e.g., Refs. [11,22]):

(7) $$\hat{H}=\sum_{m}\left[\varepsilon_{m}+\sum_{i}(\partial \varepsilon_{m}/\partial Q_{i})Q_{i}\right]{\hat{c}}_{m}^{†}{\hat{c}}_{m}+\sum_{m\neq n}\left[J_{mn}+\sum_{i}(\partial J_{mn}/\partial Q_{i})Q_{i}\right]{\hat{c}}_{m}^{†}{\hat{c}}_{n}+\sum_{i}\hbar \omega_{i}({\hat{b}}_{i}^{†}{\hat{b}}_{i}+1/2),$$

which describes an electron occupying molecular sites in an OSC and interacting with vibrational degrees of freedom. The last, vibrational term, containing phonon creation/annihilation operators ${\hat{b}}_{i}^{†}/{\hat{b}}_{i}$, was treated quasi-classically, in view of the low frequencies of the vibrational modes studied; such an LF approximation is directly related to the semiclassical transient localization framework [11,19,31,62]. 

Following a multiscale methodology, the model parameters $J_{mn},\;\omega_{i},\;\partial J_{mn}/\partial Q_{i},\partial \varepsilon_{m}/\partial Q_{i}$ for a 20 × 20 × 1 supercell were fixed by replication of the transfer integrals, vibrational frequencies, and couplings extracted from periodic DFT calculations of the NDI primitive cells, as well as from single-molecule and dimer DFT calculations (see Section 3.2 and Section 3.3). Additionally, to estimate the effect of vibrational modes beyond the Γ point on the mobility in **NDI-CPen**, periodic DFT calculations in doubled supercells were used instead of those in the primitive cell. Note that LUMO energies $\varepsilon_{m}$ are single-molecule quantities and transfer integrals $J_{mn}$ are limited to pairs of neighboring molecules with nonnegligible LUMO overlaps; both are periodic and thus can easily be copied to fill the whole transport supercell. In contrast, calculation of vibrational modes of the whole transport supercell—large enough to contain the partially delocalized charge carrier—using periodic DFT is virtually infeasible, thus, in our transport calculations, LF vibrational modes were treated as dispersionless, localized on specific molecules. For these modes, evaluated using periodic DFT in small cells and further replicated to fill the transport supercell, the couplings $\partial J_{mn}/\partial Q_{i},\partial \varepsilon_{m}/\partial Q_{i}$ were found. 

Temperature dependencies of the mobility were estimated neglecting the changes of the lattice parameters with temperature: the cell was fixed to the experimental one at 293 K. Finally, an exponential cutoff with a characteristic time $\tau_{*}=\hbar /\left(0.5\mathrm{meV}\right)$ was added to the current correlators, which is quite standard in transient-localization calculations [11,31]; the particular value of this cutoff, however, has a minor effect on the evaluated mobilities.

## 4. Conclusions

Organic semiconductors with “sparse” Raman spectra, i.e., those which consist of a limited number of bands in the LF range, deserve special attention. Namely, as the strength of the dynamic disorder correlates with the Raman LF/HF ratio and with the number of LF Raman bands, these crystals may be characterized by high charge-carrier mobilities. While performing an in silico Raman-based screening of such OSCs, however, one should be careful with the calculated frequencies and the intensities of Raman-active vibrations with wavenumbers below or around 20 cm^–1^.

In the present study, using a screening protocol based on the DFT-calculated Raman spectra, we reveal two NDI derivatives with a weak dynamic disorder. Specifically, within the series of alkyl- and cycloalkyl-substituted NDIs, compounds with cyclopentyl and butyl substituents show sparse LF Raman spectra and low LF/HF Raman intensity ratios. Subsequent charge-carrier mobility calculations using transient localization theory indeed predict very high room-temperature electron mobilities in these OSCs: up to 25 and 13 cm^2^ V^–1^ s^–1^ along certain axes in **NDI-CPen** and **NDI-But**, respectively. Thus, our study shows that screening of OSCs based on the calculated Raman spectra can be an efficient approach for searching the materials with a weak dynamic disorder and a high charge mobility, and reveals two promising OSCs for single-crystal n-type organic field effect transistors, one of them characterized by the predicted charge mobility exceeding 20 cm^2^ V^–1^ s^–1^. We anticipate that our results will facilitate the search and design of high-mobility OSC materials for organic electronics.

## Figures and Tables

**Table 1:** Selected properties of the calculated room-temperature Raman spectra (2nd–5th columns), which are relevant to the dynamic disorder amplitude; the last four columns demonstrate the two largest transfer integrals and NLEPI characteristics (*L*, σ*_J_*/*J*) in the considered OSCs. The *R* values are normalized to the one for NDI-CHex.

OSC	The Lowest-Frequency Raman-Active Band and the Most Intense Band, cm^–1 (a)^	Number of Raman-Intense Bands in the LF Region ^(b)^	Normalized LF/HF Ratio,*R/R*_CHex_	Normalized Ratio$\tilde{R}/{\tilde{R}}_{\mathrm{CHex}}$ ^(c)^	*J*_1_, meV	*J*_2_, meV	*L*, meV	*σ_J_*/*J*
**NDI-CPen**	8 (1.0); 8(1.0)	2	2.97	2.97	113	86	15	0.19
NDI-CPen *	124 (1.0); 124 (1.0)	1	0.57	0.57				
**NDI-CHex**	22 (0.52); 100 (1.0)	5	1	1	84	84	15	0.23
**NDI-CSep**	22 (1.0); 22(1.0)	7	1.11	1.10	98	43	16	0.26
**NDI-But**	63 (0.39); 118 (1.0)	7	0.72	0.68	135	46	20	0.216
**NDI-Hex**	32 (0.15); 106 (1.0)	9	1.14	1.06	90	35	31	0.402
**NDI-Una**	22 (0.73); 86 (1.0)	12	1.02	0.96	41	21	28	0.71

^(a)^ Raman intensities are given in parentheses and are normalized to the most intense band; ^(b)^ Counts the vibrations with Raman intensities above 5% of that of the most intense Raman band; ^(c)^ This ratio is based on the quantity $\tilde{R}=\lambda {\left(E_{g}-\hbar \omega_{L}\right)}^{2}R/k_{B}T$ entering the correlation in Equation (3); * This line presents the spectral characteristics calculated neglecting the mode below 20 cm^–1^.

## Data Availability

I/O files are available from the respective author upon reasonable request.

## Supplementary Materials

The following supporting information can be downloaded at: https://www.mdpi.com/article/10.3390/ijms232113305/s1. References [85,86,87,88,89,90] are cited in the supplementary materials.
