# Supplementary material for: High-Mobility Naphthalene Diimide Derivatives Revealed by Raman-Based In Silico Screening
Source: Int J Mol Sci. 2022 Nov 1;23(21):13305. doi: 10.3390/ijms232113305 (PMC9653651; doi:10.3390/ijms232113305)
Supplement: Supplementary file 1 [file ijms-23-13305-s001.zip › ijms-1991618-supplementary.pdf]

Supporting Information

High-mobility naphthalene diimide derivatives revealed by Raman-based *in silico* screening

Mikhail V. Vener [1,*], *Oleg G. Kharlanov* [2], *and Andrey Yu. Sosorev* [2,3,*]

[1] Kurnakov Institute of General and Inorganic Chemistry of the Russian Academy of Sciences, Leninskii prosp. 31, Moscow 119991, Russia

[2] Faculty of Physics, Lomonosov Moscow State University, Leninskie Gory 1/2, Moscow 119991, Russia

[3] Shemyakin–Ovchinnikov Institute of Bioorganic Chemistry of the Russian Academy of Sciences, Miklukho-Maklaya st. 16/10, Moscow 117997, Russia

S1. Details of periodic DFT calculations.

Energy tolerances controlling the self-consistent field convergence for geometry optimizations and frequency computations were set to 1×10^{-8} and 1×10^{-11} Hartree, respectively. The shrinking factor of the reciprocal space net was set to 3. Frequencies of normal modes were calculated within the harmonic approximation. Raman intensities were obtained via a coupled perturbed Hartree–Fock (CPHF) scheme [85, 86]. The number of points in the numerical first-derivative calculation of the analytic nuclear gradients equals 2.

Periodic DFT computations of molecular crystals sometimes lead to the appearance of imaginary frequencies [87, 88]. This problem is usually solved by reducing the space symmetry [89, 90]. In the present study, such procedure was applied to the **NDI-CHex** crystal computed using the B3LYP/6-31G** approximation (Table S2).

To assess the influence of the "killer mode", 2×1×1 and 1×2×1 supercells were used, formed by doubling the experimental ones of crystalline **NDI-CPen**. The experimental space group $P\bar{1}$ was used in the structural relaxation and in the calculations of Raman spectra. The computations did not reveal imaginary frequencies for any of the two supercells.

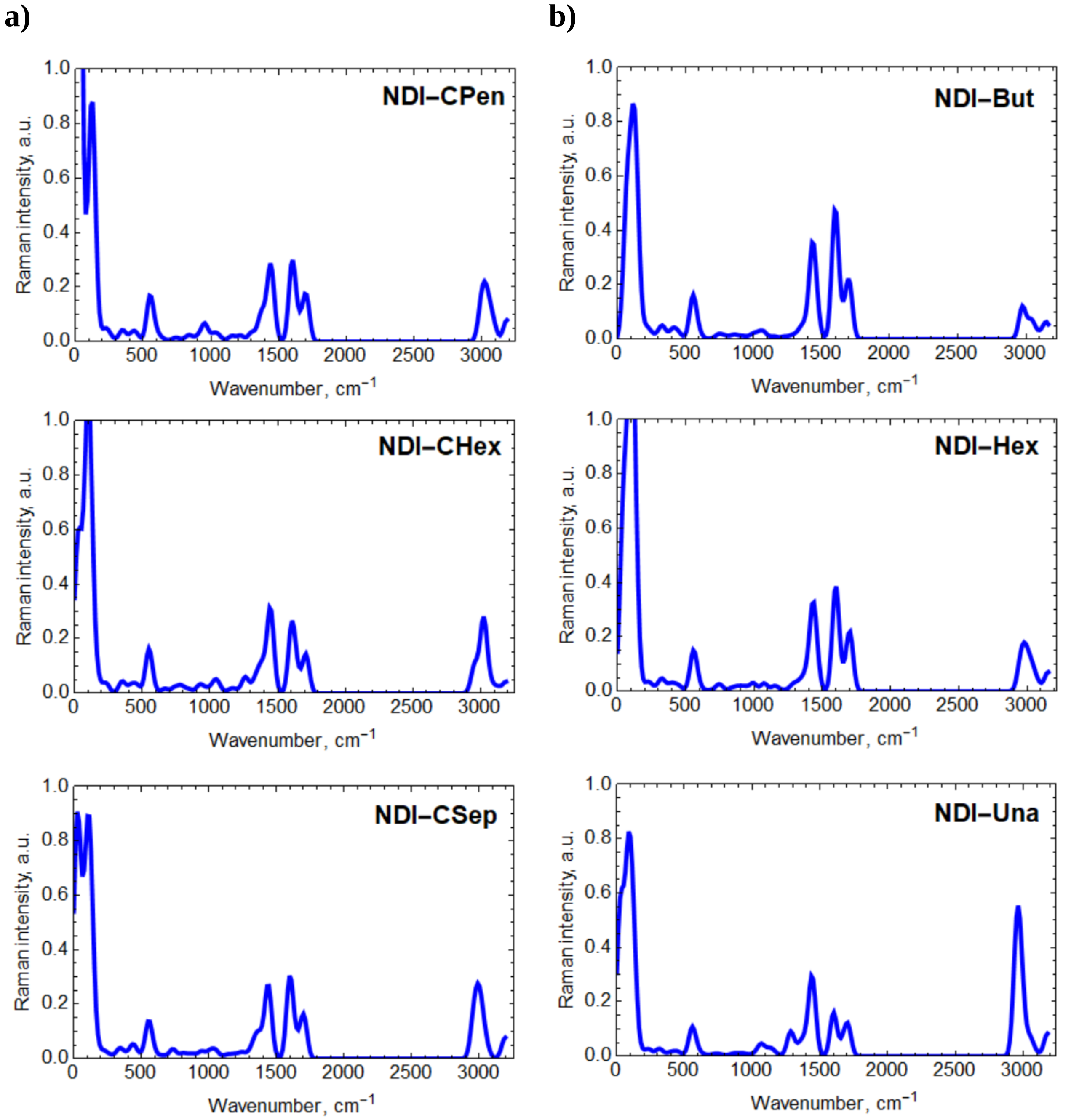

Figure S1. Theoretical Raman spectra of (a) cycloalkyl-substituted and (b) alkyl-substituted NDI crystals, calculated using DFT at the PBE-D3/6-31G** level, after Gaussian broadening with a width of 25 cm^{-1}.

Table S1. Experimental wavenumbers (cm^{-1}) of Raman-active vibrations of the **NDI-Hex** crystal [31] vs. the theoretical values found using different functionals. Relative values of Raman intensities are given in parentheses.[a)]

Exp. [b)]	Periodic DFT computations [b)]		
	PBE-D3/6-31G*	B3LYP/6-31G*	PBEsol/6-31G**
the LF region			
-	32 (0.051)	35 (0.070)	23 (0.050)
-	42 (0.050)	44 (0.084)	42 (0.363) [c)]
50 (0.235)	51 (0.449)	56 (0.457)	52 (0.050)
-			65 (0.143) [c)]
-	78 (0.134)	76 (0.064)	92 (1.000)
101 (0.360)	106 (1.000)	104 (1.000)	108 (0.103)
-	112 (0.123)	109 (0.033)	-
131 (0.176)	135 (0.179)	135 (0.104)	131 (0.227)
The medium frequency region[d)]			
559 (0.290)	557 (0.145)	571 (0.156)	558 (0.113)
600 (0.080)	-	-	-
-	1393 (0.143)	-	1405 (0.060)
1417 (1.000)	1433 (0.215) [c)]	1456 (0.408)	1455 (0.169)
1605 (0.534)	1597 (0.370)	-	1611 (0.272)
-	-	1651 (0.458)	-
1704 (0.314)	1703 (0.207)	-	1719 (0.165)

[a)] Raman intensity values given in parentheses are relative, i.e., normalized to the intensity of the most intense Raman band;

[b)] vibrations having relative Raman intensities less than 5% (0.05) are not reported;

[c)] two Raman-active bands, the frequencies of which differ by several cm^{-1}; the wavenumber of the more intense vibration of the two is given;

[d)] relative Raman intensities of vibrations in the 200–500 cm^{-1} range are quite small, therefore, these vibrations are not shown in the Table.

Table S2. Experimental wavenumbers (cm^{-1}) of Raman-active vibrations of the **NDI-CHex** crystal [31] vs. the theoretical values found using different functionalы. Relative values of Raman intensities are given in parentheses.[a)]

Exp. [b)]	Periodic DFT computations [b)]		
	PBE-D3/6-31G*	B3LYP/6-31G* [c)]	PBEsol/6-31G**
the LF region			
23 (1.000)	22 (0.505)	25 (0.980)	19 (0.441)
-	61 (0.201)	62 (0.274)	52 (0.273)
104 (0.234)	100 (1.000)	109 (1.000)	88 (1.000)
150 (0.210)	130 (0.236)	143 (0.814)	125 (0.194)
175 (0.050)	173 (0.051)	177 (0.107)	-
The medium frequency region[d)]			
551 (0.100)	550 (0.171)	562 (0.276)	550 (0.133)
790 (0.080)	-	802 (0.082);<br>1048 (0.070)	-
-	-	1293 (0.056) [e)]	-
-	1393 (0.059) [e)]	1399 (0.047)	1395 (0.081)
1426 (0.615)	1446 (0.289)	1474 (0.756)	1466 (0.191)
1608 (0.205)	1601 (0.238) [e)]	1502 (0.059)	1614 (0.185)
-	1656 (0.050)	1657 (0.453)	1638 (0.051)
1715 (0.192)	1709 (0.150)	1776 (0.265)	1725 (0.126)

[a)] Raman intensity values given in parentheses are relative, i.e., normalized to the intensity of the most intense Raman band;

[b)] vibrations having relative Raman intensities less than 5% (0.05) are not reported;

[c)] space group symmetry $P\bar{1}$;

[d)] relative Raman intensities of vibrations in the 200–500 cm^{-1} range are quite small, therefore, these vibrations are not shown in the Table;

[e)] two Raman-active bands, the frequencies of which differ by several cm^{-1}; the wavenumber of the more intense vibration of the two is given.

Table S3. Wavenumbers (cm^{-1}) of Raman-active vibrations of the **NDI** crystals computed at the PBE-D3/6-31G** level. Relative Raman intensities are given in parentheses.[a)]

cycloalkyl-substituted NDIs		alkyl-substituted NDIs	
**NDI-CPen**	**NDI-CSep**	**NDI-But**	**NDI-Una**
the LF region			
15 (1.000)	25 (1.000)	62 (0.406)	21 (0.850)
-	41 (0.213)	71 (0.431)	29 (0.591)
58 (0.053)	52 (0.252)	83 (0.202)	43 (0.121)
-	82 (0.395)	100 (0.121)	68 (0.112)
99 (0.058)	106 (0.851)	117 (0.1000)	83 (0.1000)
124 (0.496)	124 (0.652)	136 (0.106)	86 (0.516)
		143 (0.530)	94 (0.100)
			103 (0.120)
			119 (0.146)
			122 (0.717)
The medium frequency region[b)]			
552 (0.098)	555 (0.248)	557 (0.307)	556 (0.274)
-	-	-	1282 (0.176)
	1388 (0.077) [c)]	1392 (0.066)	1388 (0.076)
1439 (0.140)	1439 (0.367)	1435 (0.526) [c)]	1438 (0.342) [c)]
1599 (0.163)	1599 (0.501) [c)]	1599 (0.885)	1600 (0.417)
-	1663 (0.075)	1662 (0.112)	1667 (0.115)
1703 (0.101)	1702 (0.275)	1702 (0.398)	1706 (0.300)

[a)] Raman intensity values given in parentheses are relative, i.e., normalized to the intensity of the most intense Raman band; vibrations having relative Raman intensities less than 5% (0.05) are not reported;

[b)] relative Raman intensities of vibrations in the 200–500 cm^{-1} range are quite small, therefore, these vibrations are not shown in the Table;

[c)] Two Raman-active bands, the frequencies of which differ by several cm^{-1}; the wavenumber of the more intense vibration of the two is given.

Table S4. Raman-active LF normal modes with considerable intensities in NDI derivatives and their types.

Molecule	Intense LF Raman modes: frequencies, normalized intensities, libration axes	Atomic displacements
**NDI-CPen**	mode #4 (15 cm^{-1}),<br>intensity = 1.0,<br>short axis	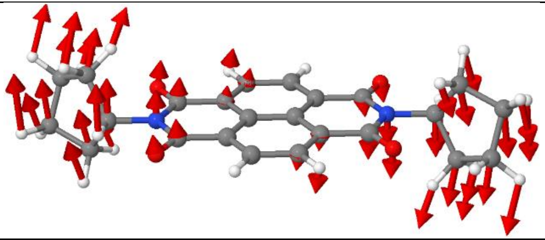
	mode #15 (124 cm^{-1}),<br>intensity = 0.50,<br>long axis	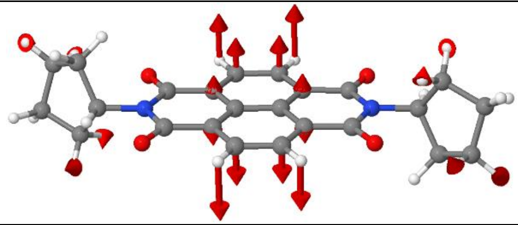
**NDI-CHex**	mode #5 (22 cm^{-1}),<br>intensity = 0.505,<br>short axis	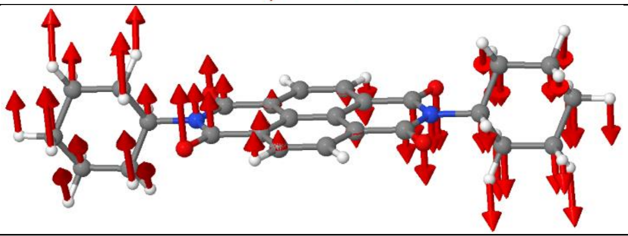
	mode #11 (100 cm^{-1}),<br>intensity = 1.0,<br>long axis	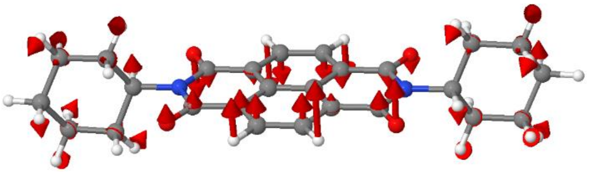
**NDI-CSep**	mode #4 (25 cm^{-1}),<br>intensity = 1.0,<br>(approx.) short axis	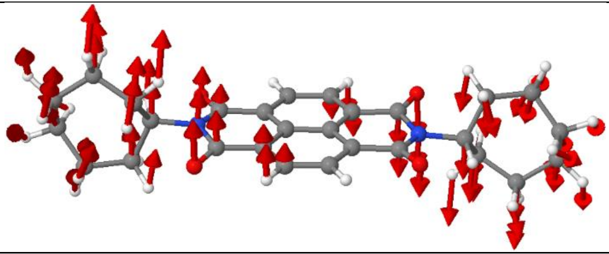
	mode #13 (106 cm^{-1}),<br>intensity = 0.85,<br>long axis	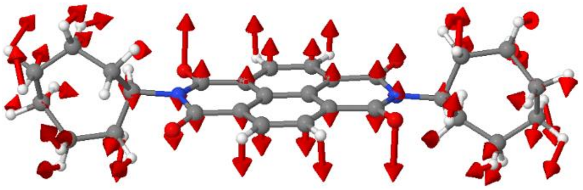
	mode #15 (124 cm^{-1}),<br>intensity = 0.65,<br>long axis	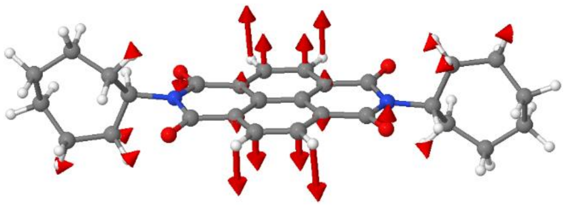

**NDI-But**	mode #4 (62 cm^{-1}), intensity = 0.41, libration axis orthogonal to the NDI core plane	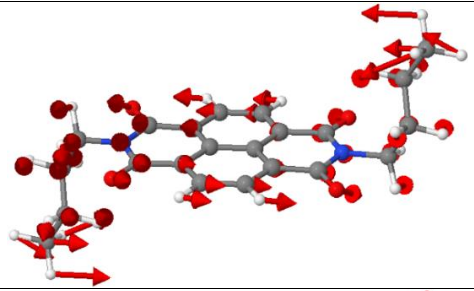
	mode #5 (71 cm^{-1}), intensity = 0.43, short axis	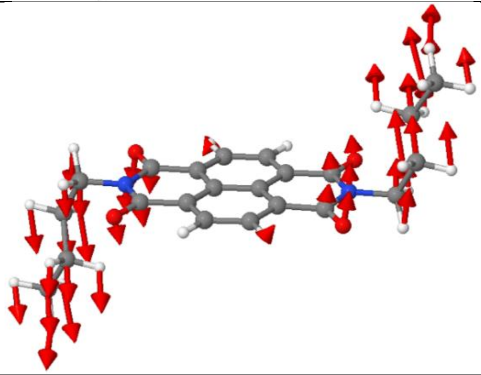
	mode #9 (83 cm^{-1}), intensity = 0.20, mixed short-long axis	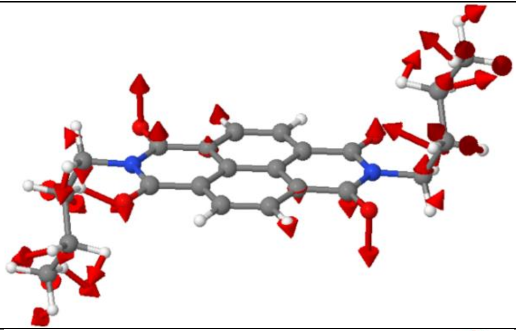
	mode #12 (117 cm^{-1}), intensity = 1.0, long axis	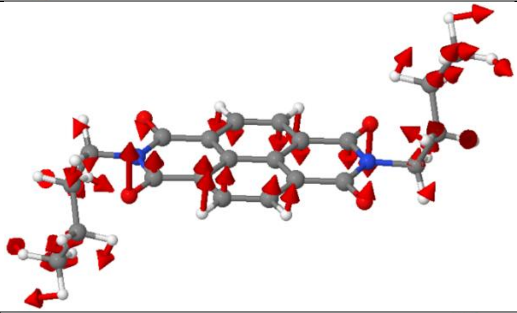
	mode #16 (143 cm^{-1}), intensity = 0.53, long axis	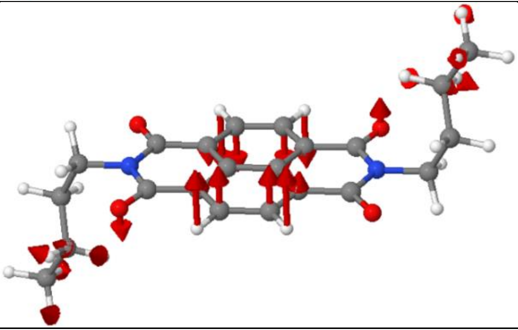

**NDI-Hex**	mode #8 (49.7 cm^{-1}),<br>intensity = 0.40,<br>(approx.) short axis	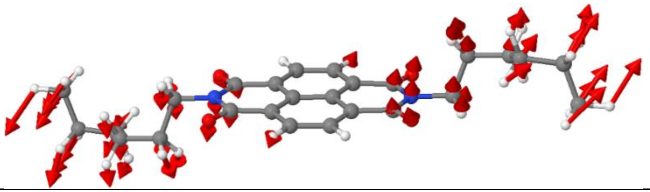
	mode #16 (105.9 cm^{-1}),<br>intensity = 1.0,<br>long axis	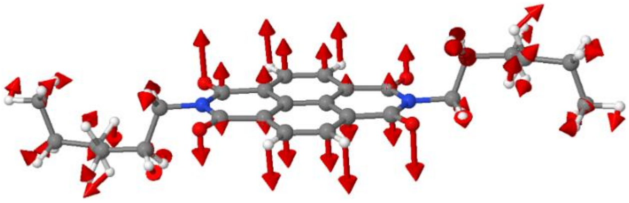
**NDI-Una**	mode #5 (21 cm^{-1}),<br>intensity = 0.85,<br>short axis	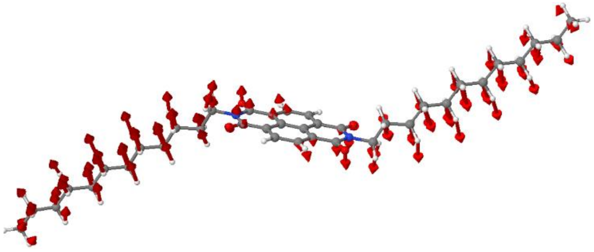
	mode #7 (29 cm^{-1}),<br>intensity = 0.79,<br>(approx.) short axis	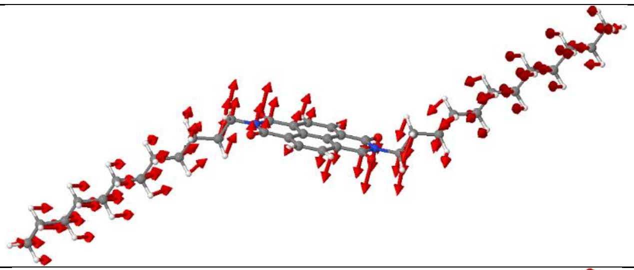
	mode #14 (83 cm^{-1}),<br>intensity = 1.0,<br>(approx.) long axis	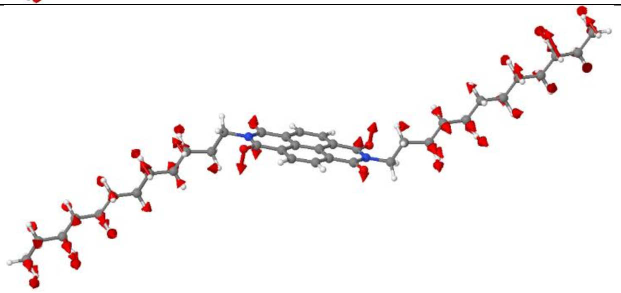
	mode #15 (86 cm^{-1}),<br>intensity = 0.52,<br>long axis	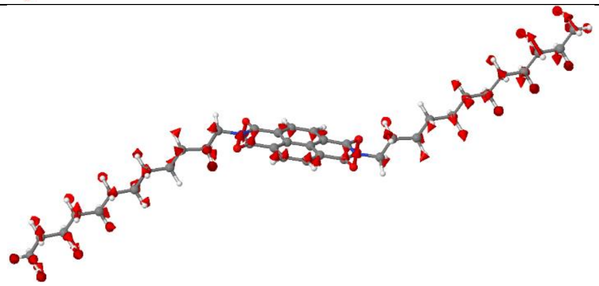
	mode #28 (122 cm^{-1}),<br>intensity = 0.72,<br>long axis	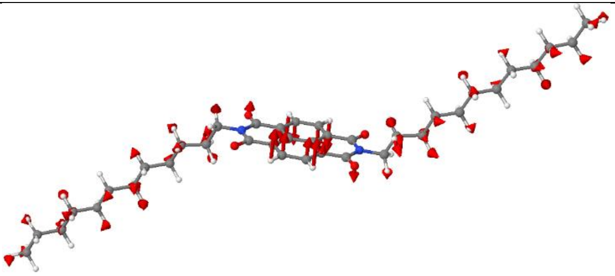

Table S5. Transfer integrals and characteristics of NLEPI in the **NDI-But** crystal (for the notations, see Sec. 3.3 of the main text).

**Direction *mn***	**J_{mn}, meV**	**L_{mn}, meV**	**$\sigma(J_{mn})$, meV**	**$\sigma(J_{mn})$ / J_{mn}**
**a**	135	15	28	0.2
**b**	46	4	14	0.31
**a+b**	19	0.2	3	0.16
total	143[a]	19.2	31[b]	0.216[c]

a) Calculated as $\sqrt{\sum_{mn} J_{mn}^2}$; b) Calculated as $\sigma_J = \sqrt{\sum_{mn} \sigma^2(J_{mn})}$;

c) Calculated as $\sigma_J/J = \sqrt{\sum_{mn} \sigma^2(J_{mn})} / \sqrt{\sum_{mn} J_{mn}^2}$.

Table S6. Transfer integrals and characteristics of NLEPI in the **NDI-CPen** crystal (for the notations, see Sec. 3.3 of the main text).

**Direction *mn***	**J_{mn}, meV**	**L_{mn}, meV**	**$\sigma(J_{mn})$, meV**	**$\sigma(J_{mn})$ / J_{mn}**
**c**	113	11	24	0.21
**a**	86	3.5	13	0.15
**a-c**	20	1	6	0.30
total	143[a]	15.5	28[b]	0.19[c]

a) Calculated as $\sqrt{\sum_{mn} J_{mn}^2}$; b) Calculated as $\sigma_J = \sqrt{\sum_{mn} \sigma^2(J_{mn})}$;

c) Calculated as $\sigma_J/J = \sqrt{\sum_{mn} \sigma^2(J_{mn})} / \sqrt{\sum_{mn} J_{mn}^2}$.

Table S7. Reorganization energies, λ, and optical gaps, E_g, for the compounds studied.

Compound	λ, meV	E_g, eV
**NDI-CPen**	358	3.207
**NDI-CHex**	360	3.203
**NDI-CSep**	361	3.197
**NDI-But**	351	3.189
**NDI-Hex**	345	3.185
**NDI-Una**	348	3.184

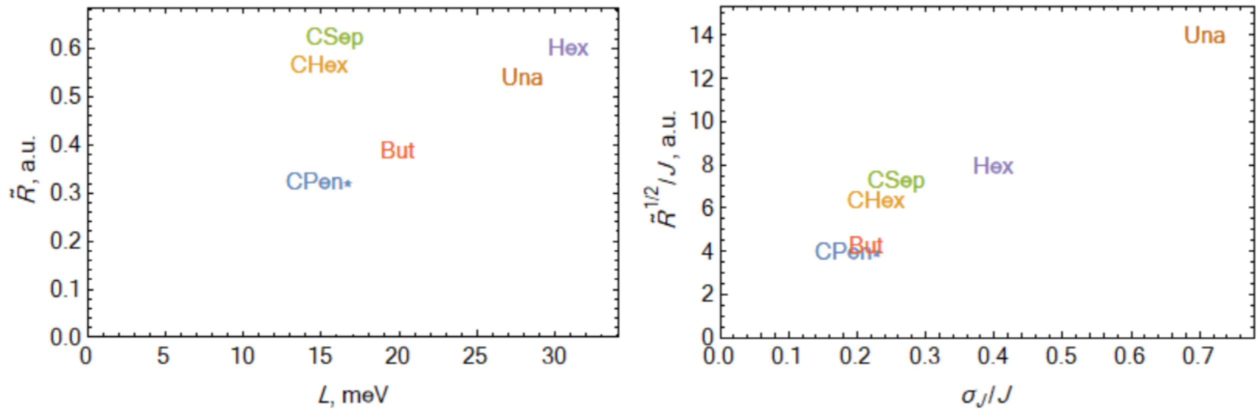

Figure S2. Correlation of the calculated ratios $\tilde{R} = \lambda\left(E_g - \hbar\omega_L\right)^2 R/k_B T$ with the lattice distortion energies, L, and the relative dynamic disorder amplitudes, σ_J/J, in the NDI derivatives studied (according to Table 1 in the main text). The figures based on the LF/HF ratio R instead of $\tilde{R}$ are almost identical to the ones shown here, because of the roughly identical energy gaps and reorganization energies in the compounds (see Table S7).

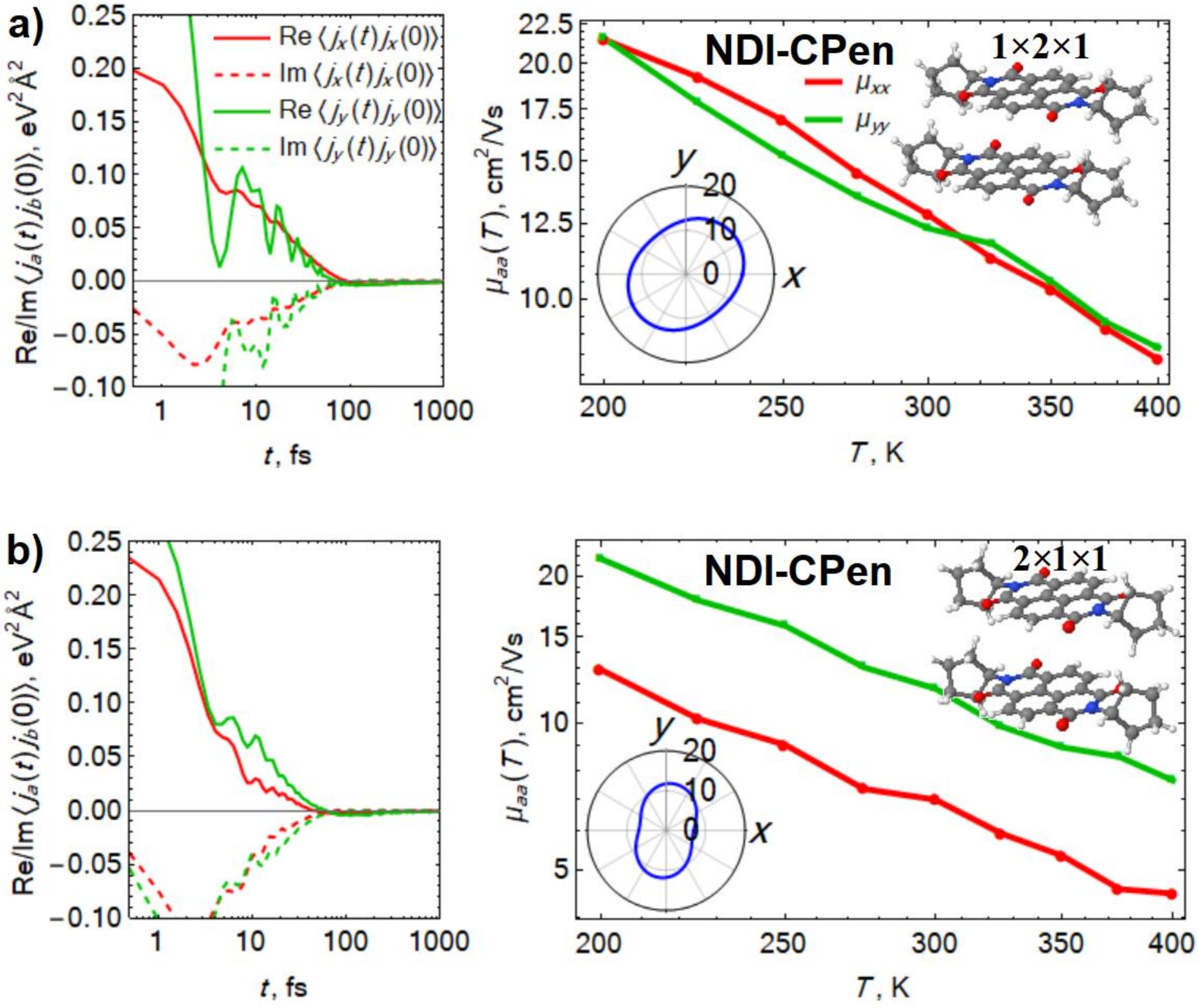

Figure S3. Room-temperature current correlators and temperature dependence of the electron mobility in **NDI-CPen** evaluated within the transient localization framework [19], starting from transfer integrals and electron-phonon couplings in a 1×2×1 supercell (a) and a 2×1×1 supercell (b). A 20×10 and a 10×20 arrays of such supercells, respectively, were used for the transport calculations.
